# Cigarette Smoking and Risk of Different Pathologic Types of Stroke: A Systematic Review and Dose-Response Meta-Analysis

**DOI:** 10.3389/fneur.2021.772373

**Published:** 2022-01-25

**Authors:** Jianyu Luo, Xiaorong Tang, Fan Li, Hao Wen, Lin Wang, Shuqi Ge, Chunzhi Tang, Nenggui Xu, Liming Lu

**Affiliations:** ^1^South China Research Center for Acupuncture and Moxibustion, Medical College of Acu-Moxi and Rehabilitation, Guangzhou University of Chinese Medicine, Guangzhou, China; ^2^Department of Biostatistics, Yale School of Public Health, New Haven, CT, United States; ^3^Center for Methods in Implementation and Prevention Science, Yale School of Public Health, New Haven, CT, United States; ^4^Department of Neurology, Sun Yat-sen Memorial Hospital, Sun Yat-sen University, Guangzhou, China

**Keywords:** stroke, cigarette smoking, dose-response, quantitative relationship, meta-analysis

## Abstract

**Objectives:**

To quantify the association of cigarette smoking, including cigarettes per day and quitting duration, with the risk of different types of stroke morbidity and mortality in the general population, and to clarify the shape of the dose-response relations.

**Study Selection:**

Prospective cohort studies and reported on the association between smoking, quitting and the incidence or mortality of stroke were included.

**Data Extraction and Synthesis:**

All available data were converted uniformly to odds ratios (ORs) and were pooled using random-effects meta-analysis with inverse variance weighting. A dose-response meta-analysis was performed to explore the quantitative relationship between different smoking characteristics and the risk of different pathologic types of stroke incidence.

**Results:**

Twenty-five studies with 3,734,216 individuals were included. Compared to never smokers, the pooled ORs of stroke morbidity and mortality were 1.45 (1.24–1.70) and 1.44 (1.23–1.67) among ever smokers and 1.90 (1.55–2.34) and 1.70 (1.45–1.98) among current smokers. The risk of different pathologic types of stroke was also increased among ever and current smokers. There was a significant non-linear dose-response association between the number of cigarette smoking and the risk of stroke incidence. Comparing no smoking, the ORs for smoking five and 35 cigarettes per day were 1.44 (1.35–1.53) and 1.86 (1.71–2.02). Other pathologic types of stroke have a similar dose-response relationship. There was also non-linear dose-response association between the length of time since quitting and risk of stroke. The risk of stroke decreased significantly after quitting for 3 years [OR = 0.56 (0.42–0.74)].

**Conclusion:**

The risk of different types of stroke among smokers is remarkably high. Our findings revealed a more detailed dose-response relationship and have important implications for developing smoking control strategies for stroke prevention.

**Systematic Review Registration:**

https://inplasy.com/inplasy-2020-6-0062/, identifier: INPLASY202060062.

## Introduction

Among 240 causes of death, stroke is the second leading cause of death and disability globally and one of the four largest contributors to disability-adjusted life years among neurological disorders (1, 2). According to the Global Burden of Disease findings in 2017, the global burden of stroke remains high, leading to 6.2 million deaths and 132.1 million disability-adjusted life-years (3). A key to reducing the global burden of stroke is renewed emphasis on stroke prevention.

Previous studies examining the association of smoking with stroke have yielded mixed findings. Smoking has been recognized as a preventable independent risk factor for stroke, with 12.4% of accidental stroke cases being attributable to current smoking behavior (4). Paradoxically, several recent studies have shown that smoking could be associated with a better early outcome in stroke patients, lower mortality rates or the same total mortality rates (5, 6). Some studies suggest that previous smoking behavior is associated with a lower clinical severity in patients with stroke (5, 7). Furthermore, smokers who received thrombolysis had a significantly greater drop in stroke severity scores from baseline than nonsmokers who received thrombolysis and lower mortality over 1 year (8, 9). However, other studies suggested that smoking was not associated with good functional outcomes after adjusting for covariates (10, 11). Given contradictory evidence in previous individual study results, additional integrative research efforts are required to reach a consensus.

Notably, the effect of smoking is closely related to its dose, and the association of different smoking characteristics with stroke warrants further investigation. A robust relationship between smoking dose and stroke can inform a decision model for doctors so that patients could possibly know how much less they need to smoke each day or how many years they need to quit smoking before experiencing noticeable health benefits. Particularly, a more precise quantification of the association between current and/or former smoking and stroke risk as well as the identification of a possible threshold for the effect remain to be determined. To date, only a few studies have examined the relationship between smoking dose and stroke and find mixed results. For example, one study used a linear model to evaluate the dose-response relationship between stroke risk and cigarette consumption (12). Other studies speculate that there is a substantial gap between this dose-response model and the actual risk of stroke (13).

Based on these existing results, we carried out a comprehensive systematic review of recent prospective cohort studies that reported the effects of smoking on the risk of different pathologic types of stroke. We evaluated the association between cigarette smoking and the risk of different pathologic types of stroke and estimate their dose-response relationship. By synthesizing evidence across studies and accounting for study heterogeneity, we characterize a more refined dose-response relationship that may have important implications for developing smoking control strategies for stroke.

## Methods

### Literature Search

For this meta-analysis, we systematically searched the PubMed, Embase, Books@Ovid, Journals@Ovid, Your Journals@Ovid, Joanna Briggs Institute EBP, ACP Journal Club, CCTR, CDSR, CCA, CLCMR, DARE, CLHTA, CLEED, AMED, Ovid Emcare, HAPI, HealthSTAR, and Ovid MEDLINE(R) databases for studies written in English and published prior to July 31, 2021.The search terms included words associated with stroke and the Cochrane Tobacco Addiction Group search strategy. The full search criteria are listed in Appendix 1. In addition, we manually searched for additional relevant articles in the reference lists of identified articles and other publications. This study follows PRISMA-IPD guidelines for individual-participant data reporting (Appendix 2). As a systematic review and meta-analysis, ethical approval was not necessary for this study. This study is registered with INPLASY (NO. INPLASY202060062).

### Inclusion and Exclusion Criteria

Articles were included if they were a prospective cohort study and provided relative risks (RRs), ORs or hazard ratios (HRs) as well as 95% confidence intervals (CIs) for the association between cigarette smoking status and stroke. Cigarette smoking status describes the status at baseline, including never and ever smoking (ever smoking includes both former smokers and current smokers). Studies that involved participants who smoked different amounts of cigarettes or who reported different lengths of time since smoking cessation were also acceptable.

Studies were excluded if they set an inexact definition of stroke or included some disease endpoints other than stroke. Compared with spontaneous intracerebral hemorrhage, traumatic hemorrhages, such as subdural and epidural hematomas and other types of intracranial bleeding that are not caused by a vascular event but due to injury, have different mechanisms, courses and outcomes. With the rapid development of technology, there is no longer a clear timeline of diagnosis between stroke and transient ischemic attack (TIA). However, they are still two different diseases because TIA leaves no permanent neurological deficit. Therefore, we only included ischemic stroke (IS) and hemorrhagic stroke (HS) (including intracerebral hemorrhage (ICH) and subarachnoid hemorrhage (SAH)). Traumatic hemorrhage and TIA should not be characterized as stroke and were not included in this meta-analysis.

We refer to the definition of stroke incidence by the American Heart Association and the American Stroke Association in 2013 and ICD codes (14). In the case of duplicate reports from the same cohort, we included the most recent publication or the publication with the longest follow-up period. Two authors (JL and XT) independently evaluated the full texts to determine whether those articles should be incorporated into the analysis. Disagreements between the two authors were settled by consensus-based discussion with a third reviewer (LL).

### Data Extraction and Quality Assessment

The follow data were extracted using a standard table: authors, year of publication, inclusion and exclusion criteria, sample size, study population (age, gender, countries and continents and whether the study patients suffer from cardiovascular disease or baseline disease or clinical information), definition of smoking, smoking status (current, former, never, dose of cigarette consumption, duration of smoking cessation), multivariate-adjusted OR, HR,or RR with 95% CIs of stroke for each smoking status category and follow-up time. This meta-analysis evaluated the correlation between cigarette smoking and the incidence or mortality of stroke, including different pathologic types of stroke, by pooling multivariate-adjusted ORs, RRs, and HRs. Multivariate adjustments were allowed to vary by study but must include age.

We evaluated the quality of the included studies using the Newcastle–Ottawa Scale (NOS) for cohort studies. Using this 9-point scale, high-quality studies were defined as a score of 7 or greater; moderate-quality studies were defined as 3–6 points; and low-quality studies were defined as below 3 points. If there were disagreements between the two authors (LW and SG) in the data extraction or quality assessment process, a third author (HW) was consulted for consensus.

### Statistical Analysis

We conducted the meta-analysis using Review Manager v.5.3 software (Cochrane Collaboration, Oxford, UK). In our study, HRs and RRs converted to ORs; HRs were considered as RRs; RRs could be converted into ORs using the formula RR = OR/[(1-P_0_) + (P_0_ × OR)], in which P_0_ was the event incidence in the control group. We converted RRs into ORs directly when studies did not provide P_0_ because the incidence or mortality of stroke in the study population is always low (<10%) (15). Multivariate-adjusted ORs of stroke with cigarette smokers (former or current) vs. never cigarette smokers were pooled by random effects models, including incidence, mortality and different pathologic types. The *I*^2^ statistic and the Cochrane Q test were used to assess between-study heterogeneity. Subgroup analysis was performed to investigate the difference between current smokers and former smokers vs. never smokers.

We performed a dose-response analysis of cigarette smoking or quitting duration on stroke risk by Stata13.1 (Stata Corporation, College Station, TX, USA). The distribution of cases, person-years and the adjusted OR with 95% CI for at least three exposure categories were required. We chose the midpoint of the interval when cigarette number or quitting duration categories intervals were presented. When the upper level for the highest category was open-ended, the exposure doses were calculated as 1.5 times their exposure levels (16). A potential non-linear dose-response association was assessed by modeling the dose and quitting duration of cigarette smoking and was checked by restricted cubic splines with four knots at the 5th, 35th, 65th, and 95th percentiles of the distribution. To test for non-linearity, a likelihood ratio test (for nested models) was applied to compare the model with both the linear and the spline terms and the model with the linear term only (17). If the non-linear model does not provide a significantly better fit for the curve, a linear model will be considered instead to quantify the association of stroke risk and cigarette consumption.

To explore factors associated with study heterogeneity, we conducted univariable random effects meta-regression for each of the outcomes when there were at least ten studies available for analysis. We also conducted a *post-hoc* multiple regression analysis adjusting for risk of stroke at baseline, type gender, length of follow-up and country. To evaluate the robustness of the results, leave-one-out sensitivity analyses were conducted for the primary outcome. We also conducted sensitivity analysis by excluding non-high-quality studies based on NOS scores.

## Results

### Search Results

**Figure 1:**
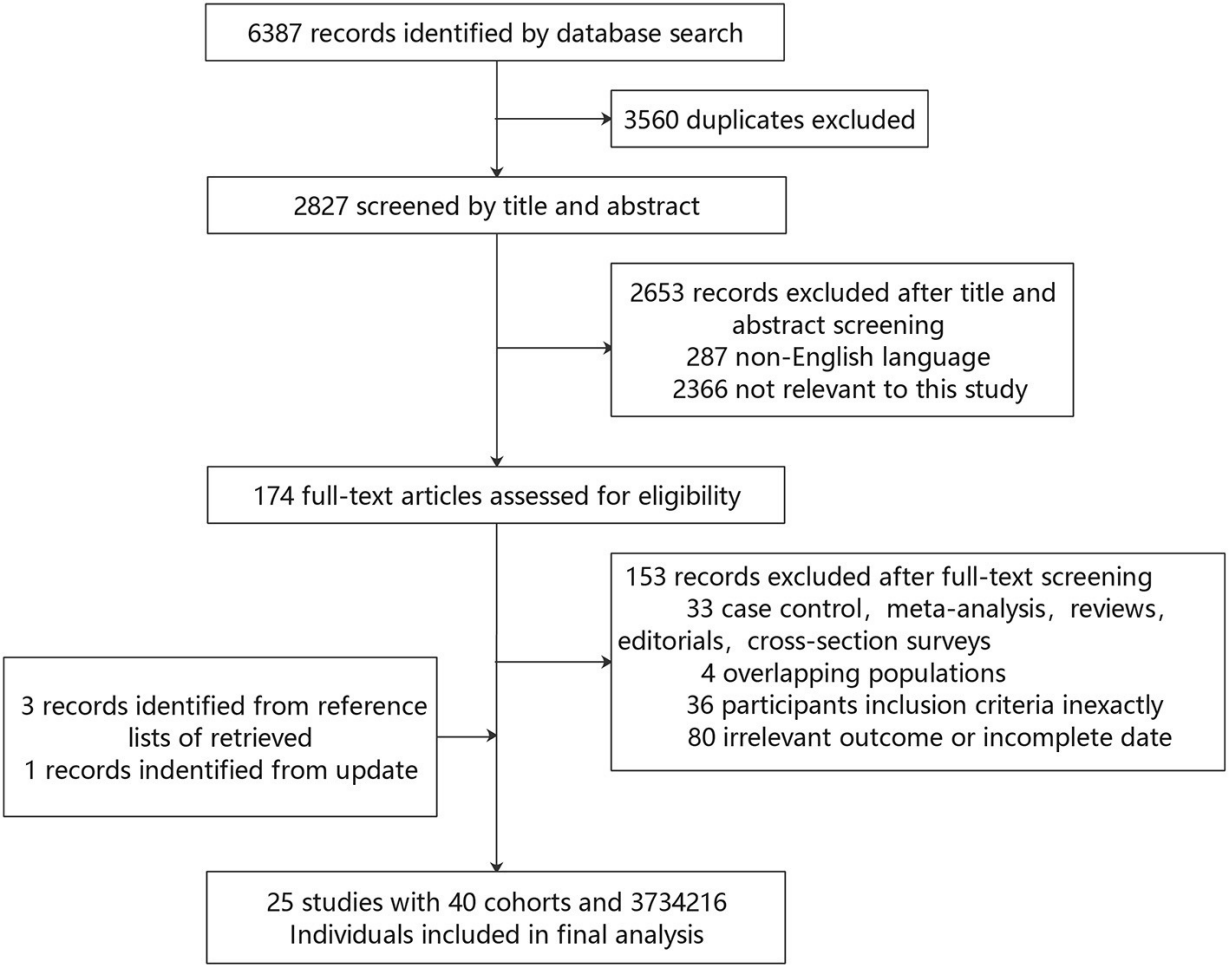
Flow diagram of the literature search.

Finally, 25 studies (18–42) were included in the meta-analysis, encompassing more than 40,000 events of stroke (Table 1). The specific screening procedure is summarized in Figure 1. Studies were from geographically diverse settings (33 countries) and the majority of the studies (88%) were rated as high quality.

**Table 1:** Characteristics of 25 prospective cohort studies of smoking and stroke events.

** 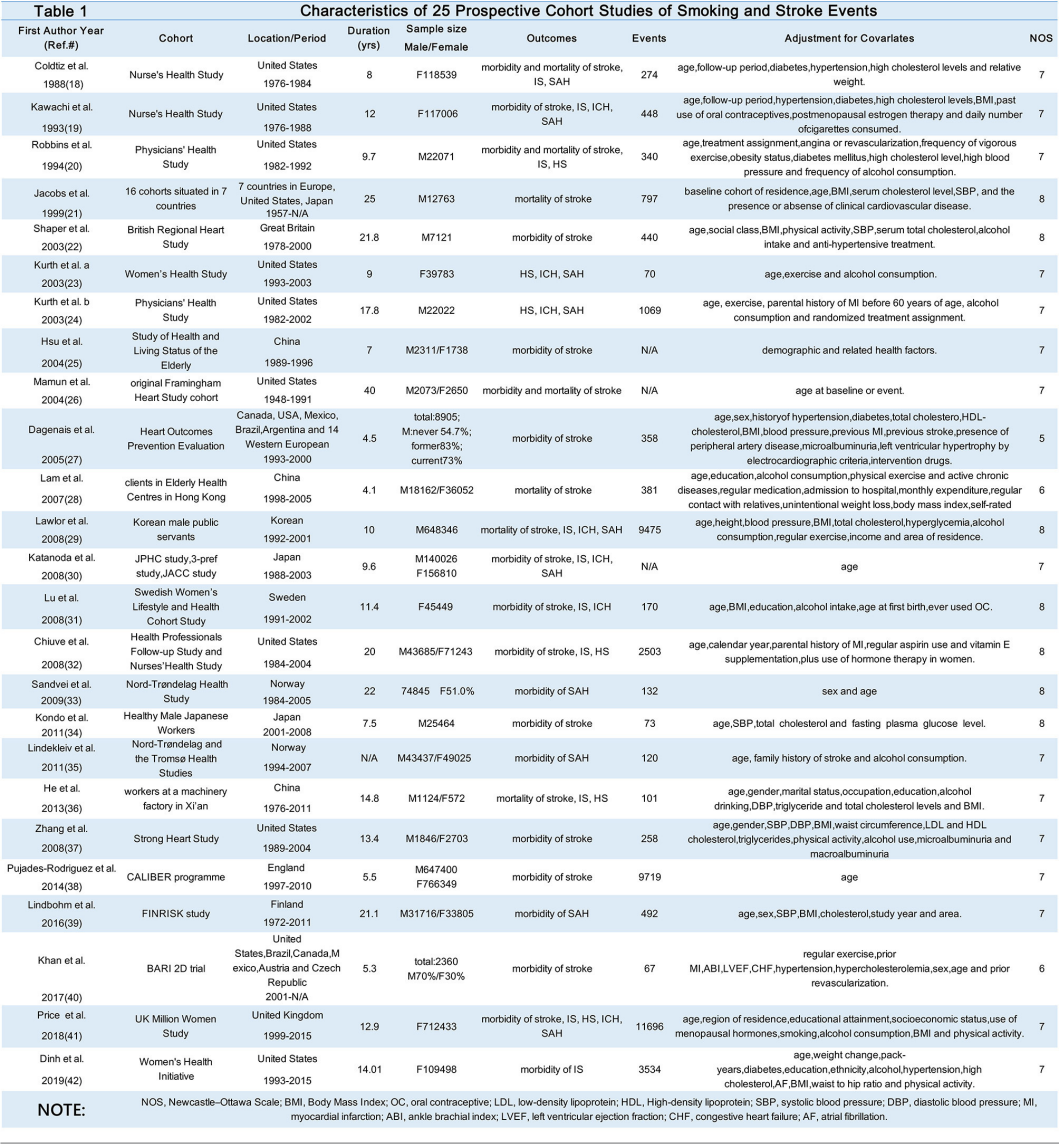 **

### Meta-Analysis

**Figure 2:**
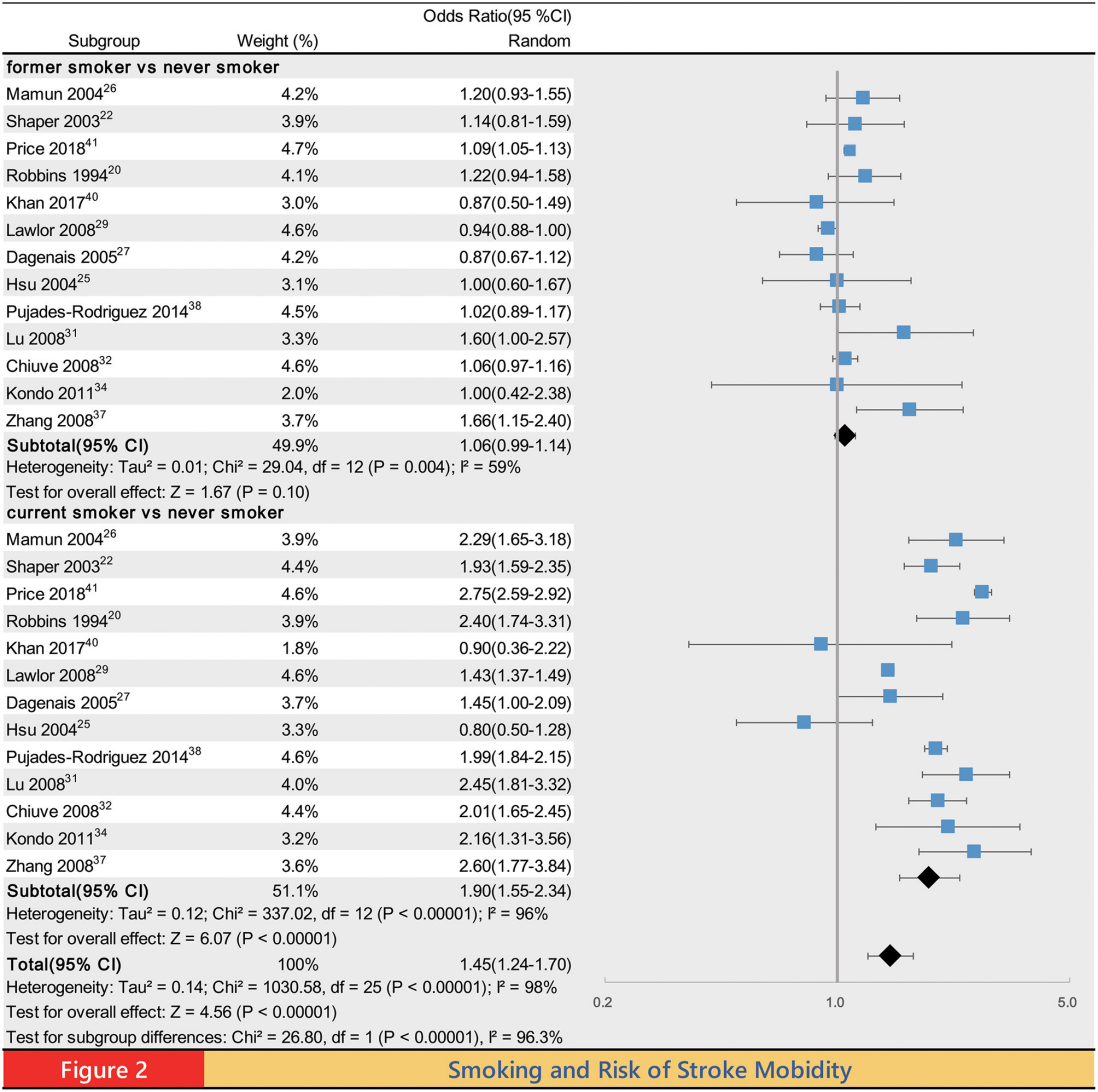
Forest plot of the primary outcome. CL, confidence interval.

Meta-analysis of the association between smoking status and stroke incidence yielded a summary OR of 1.45 (95% CI 1.24–1.70, *P* < 0.00001) for ever smokers, 1.06 (0.99, 1.14, *P* = 0.10) for former smokers and 1.90 (1.55–2.34 *P* < 0.00001) for current smokers compared with never smokers (Figure 2). Compared with never smokers, the pooled ORs of stroke mortality for ever smokers, former smokers and current smokers were 1.44 (1.23–1.67, *P* < 0.00001), 1.10 (0.99–1.22, *P* = 0.09), and 1.70 (1.45–1.98, *P* < 0.00001), respectively (Supplementary Figure 1). Compared with never smoker, the pooled OR of IS incidence for ever smokers, former smokers and current smokers was 1.55 (1.26–1.91, *P* < 0.0001), 1.05 (1.00–1.11, *P* = 0.03), and 2.09 (1.74–2.50, *P* < 0.00001; Supplementary Figure 2); the pooled OR of HS incidence for ever smokers, former smokers and current smokers was 1.49 (1.06–2.11, *P* = 0.02), 1.01 (0.86–1.18, *P* = 0.90) and 2.58 (2.23–2.97, *P* < 0.00001; Supplementary Figure 3); the pooled OR of ICH incidence for ever smokers, former smokers and current smokers was 1.25 (1.03–1.50, *P* = 0.02), 0.97 (0.84–1.13, *P* = 0.73) and 1.61 (1.17–2.23, *P* = 0.004; Supplementary Figure 4); the pooled OR of SAH incidence for ever smokers, former smokers and current smokers was 2.13 (1.60–2.85, *P* < 0.00001), 1.23 (1.02–1.49, *P* = 0.03), and 3.39 (2.59–4.45, *P* < 0.00001), respectively. Although the incidence of SAH was the lowest in the above categories of stroke (3), SAH seems to be most affected by smoking (Supplementary Figure 5).

There were statistically significant differences in the incidence of stroke, IS, HS, ICH, SAH, and mortality of stroke between ever smokers and never smokers. Such a difference was also observed between former smokers and current smokers. As expected, current smokers had the highest risk for all of these outcomes. Except for the incidence of IS and SAH, the differences between former smokers and never smokers in the risk of the rest of the outcomes were not statistically significant.

### Dose-Response Analysis

**Figure 3:**
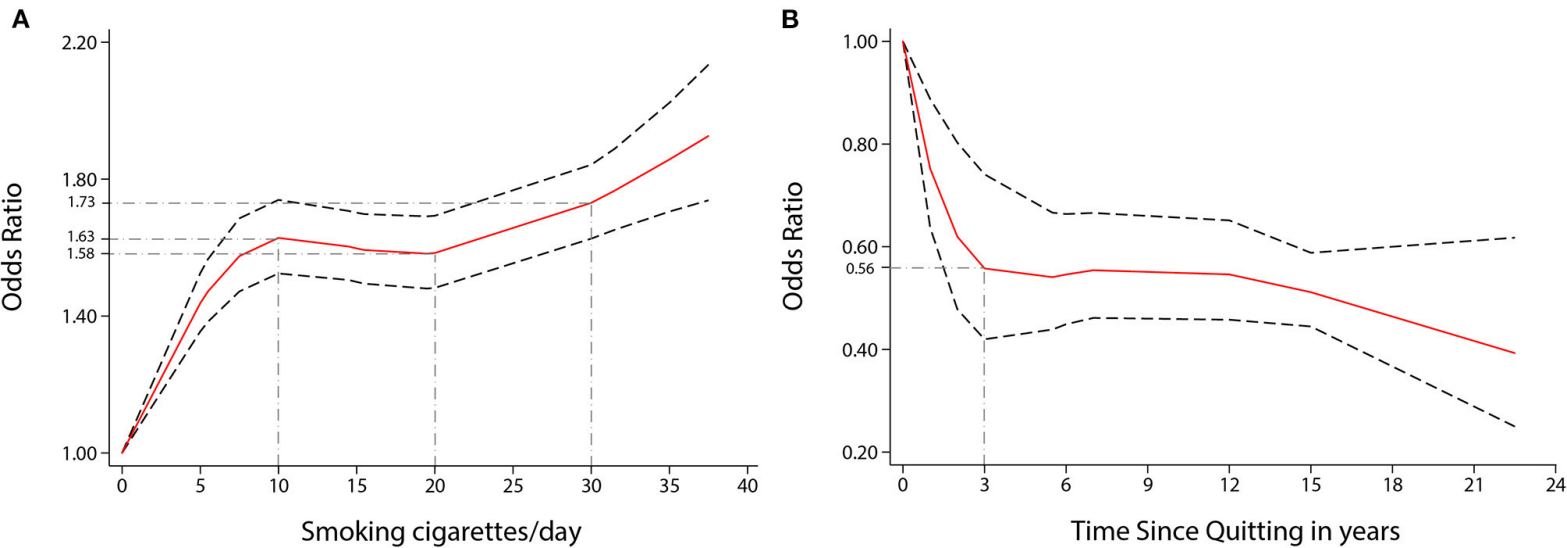
Non-linear dose-response analyses of smoking/quitting and risk of stroke in meta-analysis. **(A)** Association between CPD and risk of stroke incidence restricted cubic splines with four knots (0, 5, 15.5, 35 CPD) and 0 CPD as a reference. *P* non-linearity = 0.0000. **(B)** Association between the length of time since quitting and the risk of stroke incidence restricted cubic splines with four knots (0, 1, 6, 22.5 years) and quitting 0 years as a reference. *P* non-linearity = 0.0022. The solid line represents the estimated OR, and the dashed lines represent the 95% CI.

**Figure 4:**
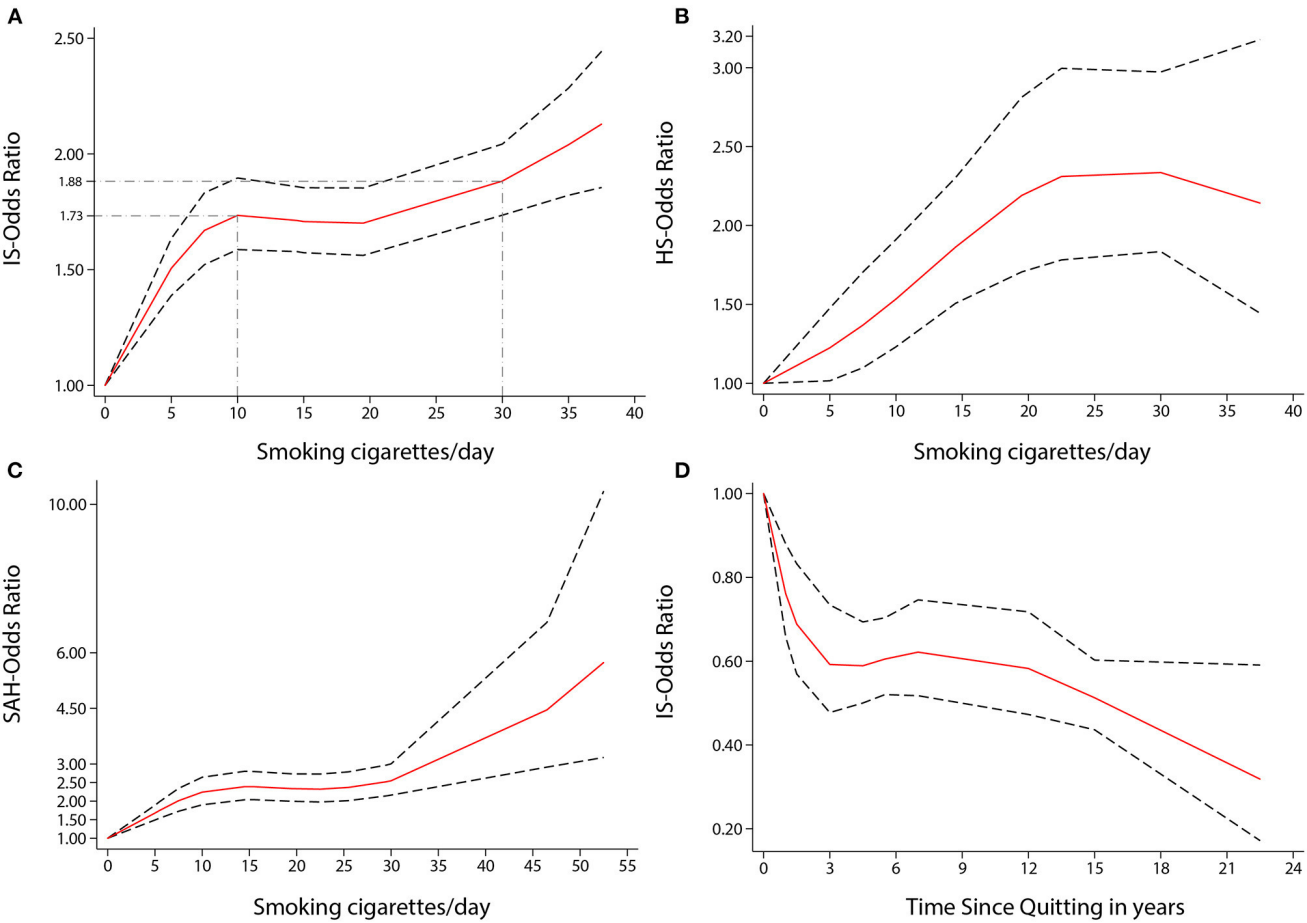
Non-linear dose-response analyses of smoking/quitting and risk of IS/HS/SAH in meta-analysis. **(A)** Association between dose of cigarette consumption and risk of Ischemic stroke incidence restricted cubic splines with 4 knots (0, 5, 15, 37.5 CPD) and 0 CPD as reference. *P* non-linearity = 0.0000. Solid line represents the estimated OR and the dashed lines represent the 95% CI. OR = 1.73, 95% CI 1.59–1.90, for 10 CPD; OR = 1.88, 95% CI 1.74–2.04, for 30 CPD. **(B)** Association between dose of cigarette consumption and risk of Hemorrhagic stroke incidence restricted cubic splines with 4 knots (0, 5, 19.5, 37.5 CPD) and 0 CPD as reference. *P* non-linearity = 0.0447. Solid line represents the estimated OR and the dashed lines represent the 95% CI. OR = 1.53, 95% CI 1.23–1.91, for 10 CPD; OR = 2.34, 95% CI 1.83–2.97, for 30 CPD. **(C)** Association between dose of cigarette consumption and risk of Subarachnoid Hemorrhage incidence restricted cubic splines with 4 knots (0, 6.5, 21, 49.5 CPD) and 0 CPD as reference. *P* non-linearity = 0.0002. Solid line represents the estimated OR and the dashed lines represent the 95% CI. OR = 2.24, 95% CI 1.90–2.64, for 10 CPD; OR = 2.55, 95% CI 2.16–3.01, for 30 CPD. **(D)** Association between the length of time since quitting and risk of Ischemic stroke incidence restricted cubic splines with 4 knots (0, 1, 5.5, 22.5 year) and quitting 0 year as reference. *P* non-linearity = 0.0022. Solid line represents the estimated OR and the dashed lines represent the 95% CI. OR = 0.59, 95% CI 0.48–0.73, for 3 year; OR = 0.51, 95% CI 0.44–0.60, for 15 year; OR = 0.32, 95% CI 0.17–0.59, for 22.5 year.

Visually, there was a significant non-linear dose-response association between the number of cigarettes per day (CPD) and risk of stroke incidence (*P* non-linearity < 0.001, Figure 3A); the OR showed a significantly increasing trend, especially as the number of CPD increased from one to ten. Compared to no smoking, the ORs for smoking five, ten, and 35 CPD were 1.44 (1.35–1.53), 1.63 (1.52–1.74), and 1.86 (1.71–2.02), respectively. Five CPD accounted for more than half of the additional risk from large doses of smoking (≥30 CPD). Smoking just ten CPD provides most of the risks of stroke associated with smoking. The OR of stroke incidence increased again when the number of CPD was more than 20. There was a similar non-linear dose-response association between CPD and risk of IS incidence (*P* non-linearity < 0.001, Figure 4A). For IS, compared to no smoking, the ORs of smoking 5, 10, and 35 CPD were 1.51 (1.39–1.63), 1.73 (1.59–1.90), and 2.04 (1.82–2.28) respectively. Similar to the stroke dose curve, smoking five CPD was associated with nearly half the additional risk from smoking in large doses (≥30 CPD).

There was a non-linear dose-response relationship between the incidence of HS and SAH and CPD. The pooled OR of incidence of HS increased rapidly when the CPD ranged from 1 to 22 (*P* non-linearity < 0.05, Figure 4B). The ORs of smoking ten and 30 CPD were 1.53 (1.23–1.91) and 2.34 (1.83–2.97), respectively. The pooled OR of SAH incidence increased rapidly when the CPD ranged from 1 to 15 and when the CPD was more than 30 (*P* non-linearity < 0.001, Figure 4C). The ORs of smoking 10 and 30 CPD were 2.24 (1.90–2.64) and 2.55 (2.16–3.01), respectively.

There was also a non-linear dose-response relationship between the incidence of stroke and the length of time since quitting cigarette smoking (*P* non-linearity < 0.01, Figure 3B). Despite only quitting for 3 years, the risk of stroke decreased rapidly [OR = 0.56 (0.42–0.74)]. Furthermore, the longer people quit smoking, the lower their risk of stroke. Similar non-linear dose-response relationship between the risk of IS incidence and the length of time since quitting [*P* non-linearity < 0.001, Figure 4D, OR = 0.59 (0.48–0.73) for three years and OR = 0.32 (0.17–0.59), for 22.5 years]. There was a tendency of stronger risk reduction for longer quitting cigarette smoking.

### Meta-Regression Analysis

We found no correlation between sex and the risk of stroke incidence (Univariable *P* = 0.265, Multiple *P* = 0.425). The continents from which people came had no correlation with the risk of stroke incidence (Univariable *P* = 0.374, Multiple *P* = 0.747). However, the follow-up time significantly modified the association between cigarette smoking and the risk of stroke incidence (Univariable *P* = 0.005, Multiple *P* = 0.013; Supplementary Table 1).

### Sensitivity Analysis

Substantial heterogeneity was observed among studies of smoking and stroke risk. However, the results of sensitivity analyses suggested that removal of any individual study did not materially alter the pooled OR; therefore, the pooled results were not dominated by any single study outlier (Supplementary Figure 6). In addition, the pooled results of sensitivity analysis in morbidity and mortality of stroke by excluding non-high-quality studies were also similar to the main results (Supplementary Figures 7, 8).

## Discussion

### Summary of Results

We have shown that smokers, especially current smokers, have a significantly increased risk of total stroke and different types of stroke, such as IS, HS, ICH, and SAH. We also show a dose-response relationship between CPD and duration of cessation and risk of different pathologic types of stroke.

Through the results of the dose-response analysis, we can clearly see that smoking and quitting smoking change stroke risk in a way that is not a simple linear relationship. Smoking has a strong and sensitive impact on stroke risk. The risk of stroke rises rapidly even with just one more CPD. While the human body's repair and adjustment functions limit the damage caused by low and medium doses of cigarette consumption, the damage caused by smoking will exceed the capacity of the human repair function when the number of CPD exceeds 20. There is another conjecture that explains such an association: when the number of CPD exceeds 20, different damage mechanisms in the body appear or dominate, so the risk of stroke rises rapidly again.

According to the results of the dose-response analysis of quitting, we found that the risk of stroke and IS drop rapidly in the first three years of quitting. This means that the tendency to decrease thrombosis and cerebral perfusion and the negative effects on changes in hemodynamics function and thrombosis caused by smoking might be reversed by the third year. Atherosclerosis from smoking can also be repaired by the human themselves, but it takes more than ten years to show up in a reduction of the stroke risk, and the longer the time, the greater the effect. The risk difference between former and never smokers was not statistically significant in the incidence of stroke (*p* = 0.10), HS (*p* = 0.90), ICH (*p* = 0.73) or the mortality of stroke (*p* = 0.09) in the subgroup analysis. Therefore, the effects of smoking on the risk of stroke may be reversed by the body's strong repair ability.

### Mechanism of Smoking and Stroke

The deleterious effect of cigarette smoke is related to a mixture of more than 7000 chemicals contributing to endothelial dysfunction, inflammation, dyslipidemia, vascular and hemodynamic function, and a prothrombotic state. This can cause atherosclerosis and increase the risk of thrombotic events. Decreased vasodilatation and diminished nitric oxide bioavailability were also observed in smokers (43). The effects of the above causes and mechanisms greatly increase the risk of cardiovascular disease. Cerebrovascular disease has a similar pathogenesis. Atherosclerosis formation, thrombosis and decreased cerebral perfusion increase the risk of stroke, especially IS. Kurth et al. (23) summarized that smoking increased the risk of SAH by promoting the presence, formation and rupture of aneurysms and increased the risk of ICH by damaging the structure of the arterial wall.

### Study Strengths and Limitations

Our study used a nonlinear model to demonstrate a more realistic dose-response relationship, and facilitate a accurate understanding of the relationship between smoking dosage and risk of stroke. In addition, we further refined the dose-response relationship between each stroke type (IS, HS, ICH, and SAH) and the number of cigarettes smoked. We also dynamically analyzed the relationship between length of smoking cessation and risk reduction, which could provide clearer guidance and stronger confidence to quitters and potential quitters. Moreover, we limited the ICD codes of the included study and removed age restrictions for a more comprehensive analysis of stroke. Compared to previously published studies, our study included more detailed analyses to support our findings, which leads to new insights.

The most important point among these factors was that we observed a significant nonlinear dose-response association between CPD and quitting and the risk of each stroke type (IS, HS, ICH and SAH) incidence. Hackshaw et al. (12) used a log-linear variance weighted regression model to evaluate the dose-response relationship between stroke risk and cigarette consumption. They consider that smoking one CPD had 41% and 31% of the excess RR of men and women who smoked 20 CPD, respectively. However, we came up with inconsistent results. Although we agree that small CPD also poses a significant risk of stroke, smoking only five CPD led to more than half of the additional risk from 20 or 30 CPD, and ten CPD provided most of the risk of stroke associated with smoking. However, it is unreasonable that one cigarette brings approximately half of the excess risk of one pack of cigarettes. Their model may have exaggerated the risk of smoking one cigarette and 20 cigarettes according to our results. In addition, Oono et al. (44) also used a nonlinear dose-response relationship, but they only discussed the relationship between second-hand smoke and stroke.

It is also worth mentioning that part of the stroke cohort studies that did not use accurate stroke definitions and ICD codes, equated stroke directly with cerebrovascular diseases (ICD9, 430-438, ICD10, I60-I69). Some of them may also include TIA. All previous meta-analyses on smoking and stroke did not exclude research with inaccurate definitions. This led to an inaccurate number of stroke events and an overestimation of stroke risk. Our study set the inclusion criteria to bring our results closer to the real relationship between smoking and stroke risk.

Furthermore, Mons et al. (45) reported a lower risk of stroke from smoking. That was because they included older people over the age of 60. The reason why smoking has a less negative effect is that older people have a higher incidence and mortality of stroke. However, stroke should no longer be considered a disease of the elderly, and two-thirds of all strokes occur among persons <70 years of age reported by Global Burden Disease study (46). Krishnamurthi et al. (3) also found a concerning trend toward increased stroke burden in people aged 45–59 years old. Therefore, our inclusion criteria did not limit the age of participants, and our outcome applies to a wider range of populations.

This study has several limitations. Because some articles were excluded due to inexact definitions of stroke and ICD codes, the data related to smoking and outcomes for IS, ICH, and SAH mortality are sparse. We were unable to assess dose-response analysis between smoking, quitting and the mortality of different pathologic types of stroke.

In addition, heterogeneity across studies was high among the included studies and may be due to different study designs and characteristics of participants. For example, the sample size, follow-up time, multiple adjustments and definitions of never, former and current smokers varied widely from study to study. However, sensitivity analyses showed that any meta-analysis did not alter the pooled OR significant, and the pooled results were stable.

### Policy Implications

Due to its high morbidity, mortality and disability rate, stroke has brought a heavy burden to modern society in different aspects. At present, the stroke population is still increasing, for example in China, and carrying out primary stroke prevention, such as reducing smoking, is the best way to prevent stroke (47). Therefore, the government and media need to publicize tobacco control in more detail and quantitatively to make it more effective. Health professionals and the public should realize that low-dose (five to ten CPD) cigarette consumption is associated with a high risk of stroke, and cessation for more than three years is associated with significant benefits. If people cannot quit smoking, they should be limited to five or fewer CPD to significantly reduce their risk of stroke. In addition, the government should also encourage and support the development of smoking cessation institutions. Moreover, smoke-free laws should be enforced in public places and indoors to reduce exposure to cigarette smoking.

## Conclusion

Smoking will increase the risk of stroke with different pathologic types. There was a non-linear dose-response relationship between the amount of cigarette smoking and duration of cessation and stroke risk. Low-dose smoking can carry half or more of the additional risk from large doses of smoking. Quitting smoking for more than 3 years will deliver significant health benefits. Our findings provide a more detailed dose-response relationship and have important implications for developing smoking control strategies for stroke.

## Data Availability Statement

The raw data supporting the conclusions of this article will be made available by the authors, without undue reservation.

## Author Contributions

LL developed the study concept. JL and XT did the literature search, article screening, data extraction, and statistical analyses. HW, LW, and SG evaluated the quality of studies. FL, CT, NX, and LL directed research and revised manuscripts. All authors were involved in drafting the manuscript.

## Funding

This work was supported by the Key-Area Research and Development Program of Guangdong Province (2020B1111100008 to LL), the Youth Scientific Research Training Project of GZUCM (2019QNPY02 to LL), the Young Top Talent Project of Scientific and Technological Innovation in Special Support Plan for Training High-level Talents in Guangdong (2017TQ04R627 to LL), the Youth Program of the National Natural Science Foundation of China (81904297 to LW), the Elite Youth Education Program of Guangzhou University of Chinese Medicine (QNYC20190106 to LW), and the Science and Technology Planning Project of Guangdong Province (2014B090902002 to NX). The funders of the study had no role in study design, data collection, data analysis, data interpretation, or writing of the report.

## Conflict of Interest

The authors declare that the research was conducted in the absence of any commercial or financial relationships that could be construed as a potential conflict of interest.

## Publisher's Note

All claims expressed in this article are solely those of the authors and do not necessarily represent those of their affiliated organizations, or those of the publisher, the editors and the reviewers. Any product that may be evaluated in this article, or claim that may be made by its manufacturer, is not guaranteed or endorsed by the publisher.
