# Supplementary material for: Cigarette Smoking and Risk of Different Pathologic Types of Stroke: A Systematic Review and Dose-Response Meta-Analysis
Source: Front Neurol. 2022 Jan 25;12:772373. doi: 10.3389/fneur.2021.772373 (PMC8821532; doi:10.3389/fneur.2021.772373)

**Supplementary Appendix**

**Cigarette Smoking and Risk of Different Pathologic Types of Stroke: A Comprehensive Systematic Review and Dose-response Meta-analysis**

**Catalogue**

[**Appendix 1： Search strategy 3**](#_Toc11498)

[**Online Figure 1： Forest plot of cigarette smoking on stroke mortality in meta-analysis 5**](#_Toc17963)

[**Online Figure 2： Forest plot of cigarette smoking on Ischemic Stroke in meta-analysis 6**](#_Toc3418)

[**Online Figure 3： Forest plot of cigarette smoking on Hemorrhagic Stroke in meta-analysis 7**](#_Toc2792)

[**Online Figure 4： Forest plot of cigarette smoking on Intracerebral Hemorrhage in meta-analysis 8**](#_Toc13602)

[**Online Figure 5： Forest plot of cigarette smoking on Subarachnoid Hemorrhage in meta-analysis 9**](#_Toc14646)

[**Online Table 1: univariable and multiple regression analysis for the risk of stroke morbidity 10**](#_Toc8800)

[**Online Figure 6: leave-one-out sensitivity analyses for the risk of stroke morbidity 11**](#_Toc19875)

[**Online Figure 7: sensitivity analysis by excluding non-high-quality studies for Stroke morbidity 12**](#_Toc709)

[**Online Figure 8: sensitivity analysis by excluding non-high-quality studies for stroke mortality 13**](#_Toc17904)

[**Online Figure 9: Definitions of Former Smoker in Meta-analysis 14**](#_Toc30226)

# Appendix 1： Search strategy

Search strategy of the PubMed, Embase, Books@Ovid, Journals@Ovid, Your Journals@Ovid, Joanna Briggs Institute EBP Database -, ACP Journal Club, CCTR, CDSR, CCA, CLCMR, DARE, CLHTA, CLEED, AMED, Ovid Emcare, HAPI, HealthSTAR, Ovid MEDLINE(R) by using Ovid.

1. (stroke$ or poststroke or apoplex$ or cerebral vasc$ or brain vasc$ or cerebrovasc$ or cva$ or SAH).tw.

2. exp brain h?ematoma/ or exp carotid artery disease/ or exp cerebral artery disease/ or exp cerebral small vessel disease/ or exp cerebrovascular accident/ or exp intracranial aneurysm/ or exp brain hemangioma/ or exp moyamoya/ or exp lacunar stroke/ or exp vertebrobasilar insufficiency/ or exp vasospasm/

3.((brain$ or cerebr$ or cerebell$ or intracerebral or intracran$ or parenchymal or intraparenchymal or intraventricular or brainstem or infratentorial or supratentorial or basal gangli$ or putaminal or putamen or posterior fossa or hemispher$ or subarachnoid) adj5 (h?emorrhag$ or h?ematoma$ or bleed$)).tw.

4.((brain or cerebr$ or cerebell$ or vertebrobasil$ or hemispher$ or intracerebral or intracran$ or brainstem or infratentorial or supratentorial or middle cerebral artery or MCA$ or anterior circulation or posterior circulation or basilar artery or vertebral artery or space occupying) adj5 (isch?emi$ or infarct$ or thrombo$ or emboli$ or occlus$ or hypoxi$)).tw.

5.(hemipleg$ or hemipar$ or paresis or paretic or paralysis).tw.

6.1 or 2 or 3 or 4 or 5

7.exp Prospective-Studies/

8.exp Evaluation-Studies/ or Program-Evaluation.mp.

9.exp Cross-Sectional-Studies/

10.Comparative study/

11.exp Behavior-therapy/

12.exp Health-Promotion/

13.exp Community-Health-Services/

14.exp Health-Behavior/ or exp Health-Education/

15.exp Vital Statistics/

16.exp epidemiology/

17.7 or 8 or 9 or 10 or 11 or 12 or 13 or 14 or 15 or 16

18.smoking cessation.mp. or exp Smoking Cessation/

19.tobacco cessation.mp. or "Tobacco-Use-Cessation"/

20.exp Smoking/

21."Tobacco-Use-Disorder"/

22.Tobacco-Smokeless/

23.exp Tobacco-Smoke-Pollution/

24.Smoking reduction/ or Smoking reduction.mp.

25.Smoking prevention/

26. Vaping/ or vaping.mp.

27. Electronic Nicotine Delivery Systems/

28. electronic cigar*.mp.

29. exp Pipe smoking/ or exp Tobacco smoking/ or exp Tobacco Products/

30. ((quit$ or stop$ or ceas$ or giv$ or abstain* or abstinen*) adj5 (smoking or smoke* or tobacco)).ti,ab.

31.exp Tobacco/ or exp Nicotine/

32.18 or 19 or 20 or 21 or 22 or 23 or 24 or 25 or 26 or 27 or 28 or 29 or 30 or 31

33.32 and 17

34.6 and 33

35.(humans not animals).sh.

36.34 and 35

# Online Figure 1： Forest plot of cigarette smoking on stroke mortality in meta-analysis

# Online Figure 2： Forest plot of cigarette smoking on Ischemic Stroke in meta-analysis

# Online Figure 3： Forest plot of cigarette smoking on Hemorrhagic Stroke in meta-analysis

# Online Figure 4： Forest plot of cigarette smoking on Intracerebral Hemorrhage in meta-analysis

# Online Figure 5： Forest plot of cigarette smoking on Subarachnoid Hemorrhage in meta-analysis

**Online Table 1: univariable** **and multiple regression analysis for the risk of stroke morbidity**

| **comparison** | odds ratio（95% CI） | P value | |
| --- | --- | --- | --- |
|  |  | Univariable  meta-regression | Multiple  meta-regression |
| subgroup by continental |  |  |  |
| North America | 1.213（1.134 to 1.291） | 0.374 | 0.747 |
| Europe | 1.456（1.415 to 1.498） |  |  |
| Asia | 1.254（1.210 to 1.298） |  |  |
| subgroup by female sex ratio |  |  |  |
| ＜25% | 1.282（1.238 to 1.326） | 0.265 | 0.425 |
| 25%-75% | 1.335（1.270 to 1.400） |  |  |
| ＞75% | 1.411（1.366 to 1.456） |  |  |
| subgroup by follow years |  |  |  |
| ＜10 | 1.301（1.261 to 1.341） | 0.005 | 0.013 |

**Online Figure 6: leave-one-out sensitivity analyses** **for the risk of stroke morbidity**


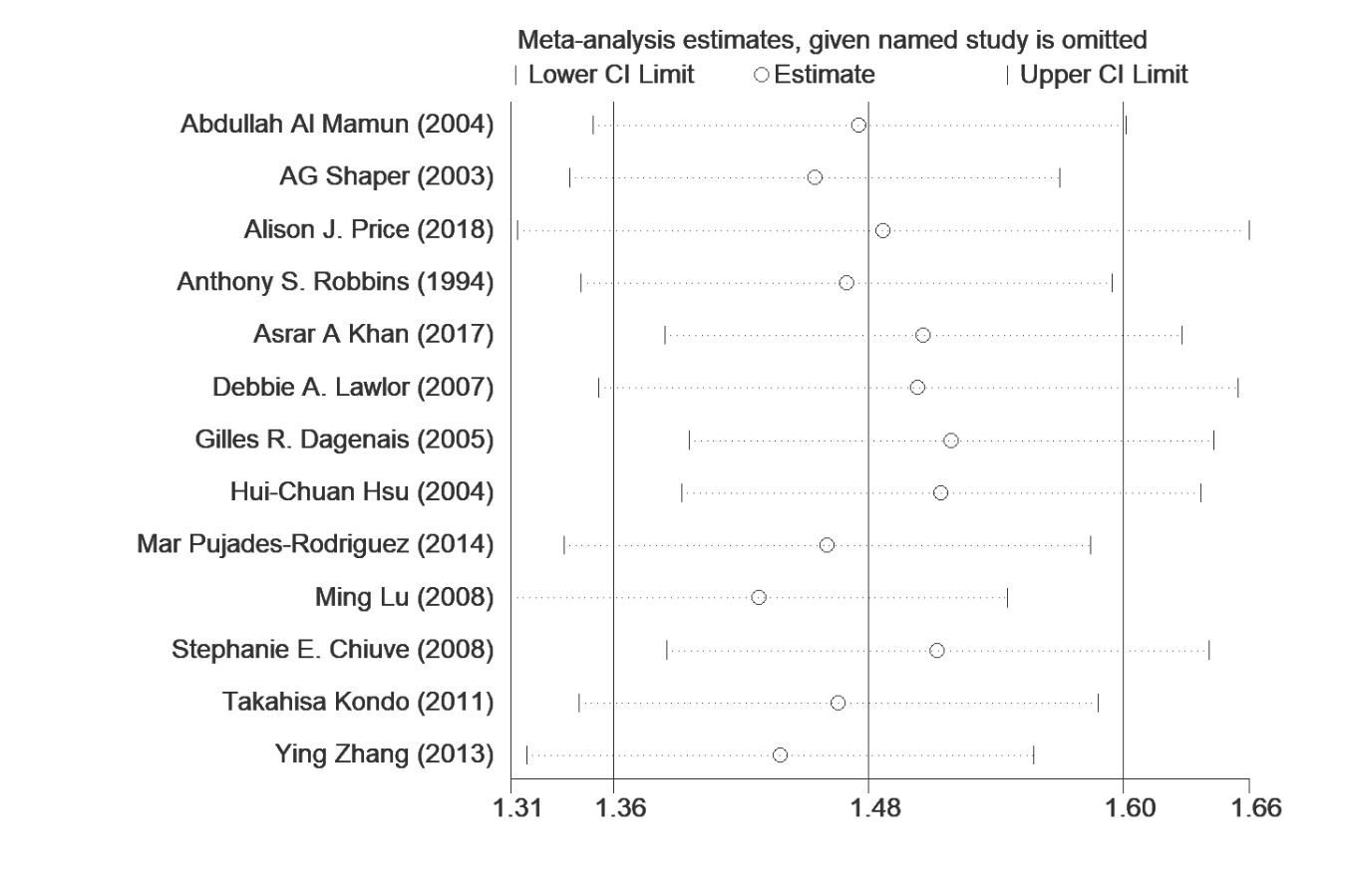


**Online Figure 7: sensitivity analysis by excluding non-high-quality studies for Stroke morbidity**

**
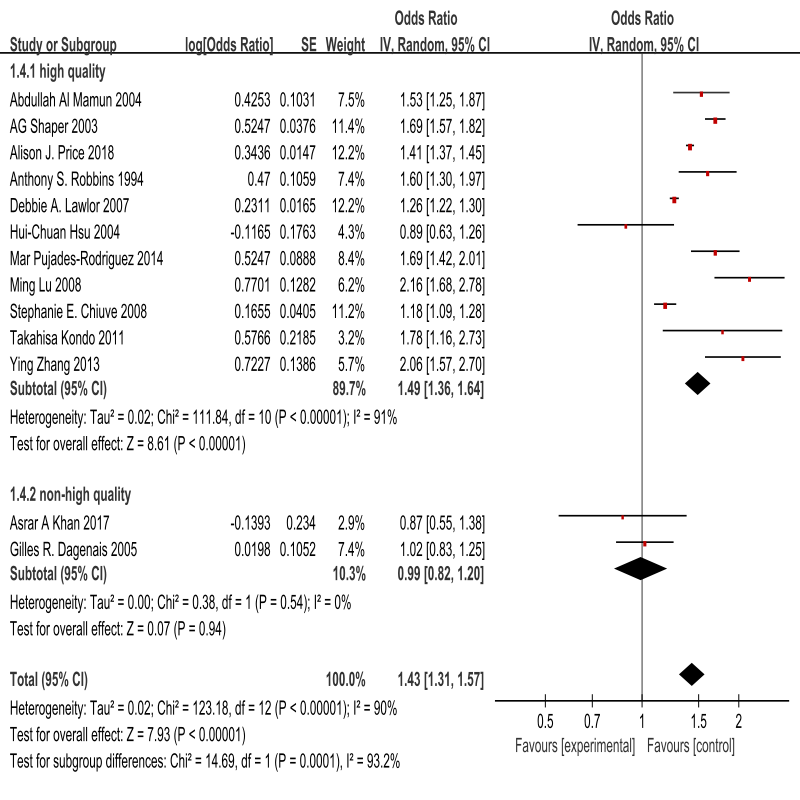
**

**Online Figure 8: sensitivity analysis by excluding non-high-quality studies for stroke mortality**


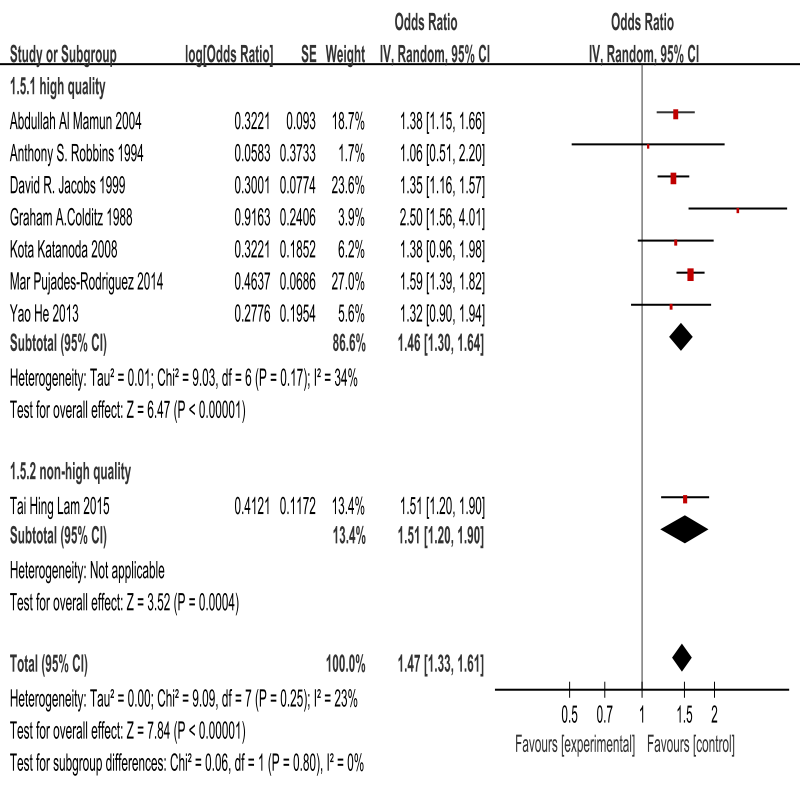


**Online Figure 9: Definitions of Former Smoker in Meta-analysis**


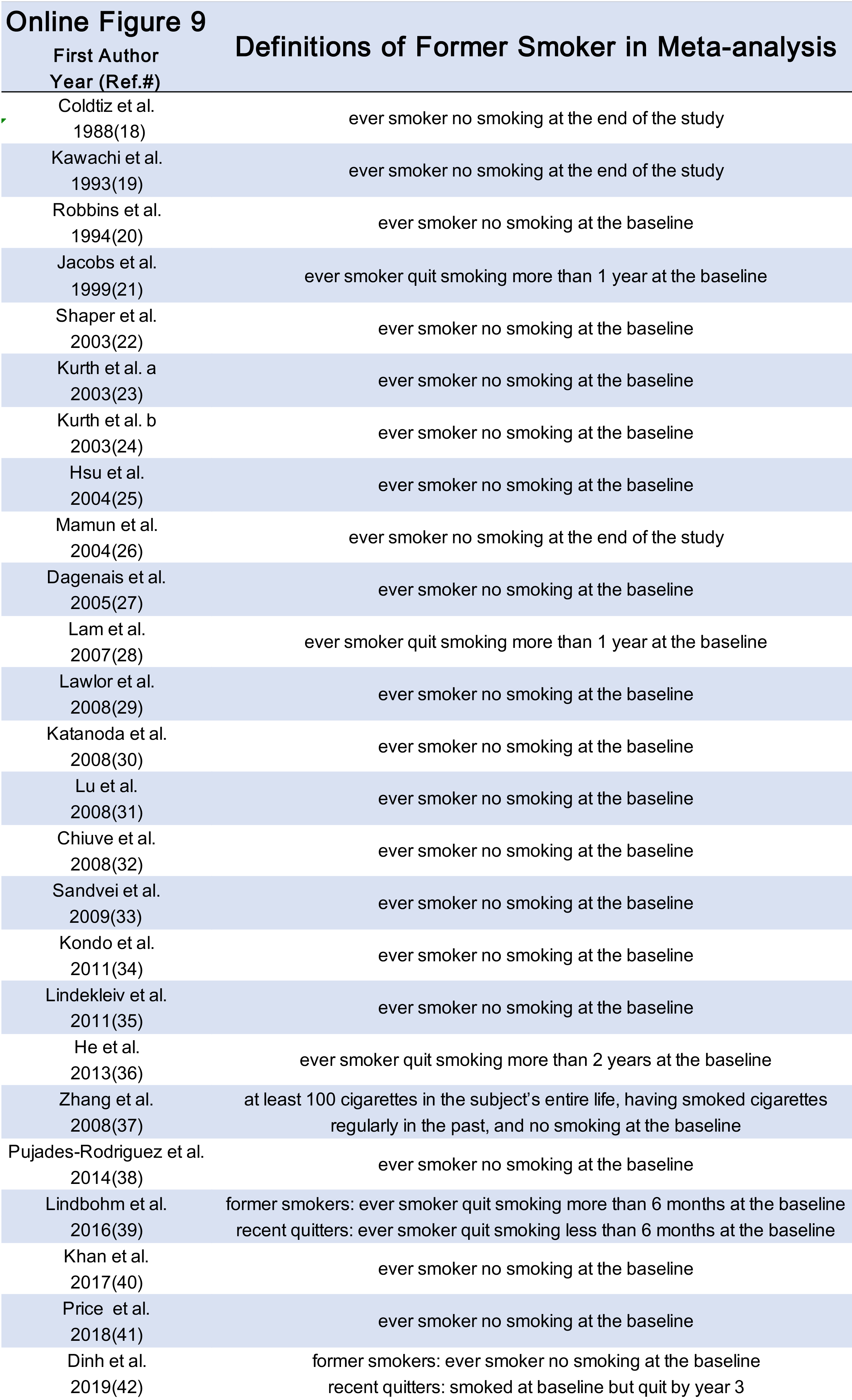

Supplement: Supplementary file 1 [file Data_Sheet_1.docx]
